# A cluster-randomized controlled trial to study the effectiveness of a protocol-based lifestyle program to prevent type 2 diabetes in people with impaired fasting glucose

**DOI:** 10.1186/1471-2296-14-184

**Published:** 2013-12-02

**Authors:** Arlette E Hesselink, Henk JG Bilo, Ruud Jonkers, Marloes Martens, Inge de Weerdt, Guy EH Rutten

**Affiliations:** 1 ResCon, Research & Consultancy, Haarlem, Netherlands; 2 Diabetes Centre, Isala clinics and Department of Internal Medicine, Isala Clinics, Zwolle; and, University Medical Centre Groningen, Groningen, Netherlands; 3 Dutch Diabetes Federation, Amersfoort, Netherlands; 4 Julius Center for Health Sciences and Primary Care, Utrecht, Netherlands

**Keywords:** Diabetes, Prevention, Healthy life style intervention, Impaired fasting glucose, Primary care health services, Randomized clinical trial

## Abstract

**Background:**

Effective diabetes prevention strategies that can be implemented in daily practice, without huge amounts of money and a lot of personnel are needed. The Dutch Diabetes Federation developed a protocol for coaching people with impaired fasting glucose (IFG; according to WHO criteria: 6.1 to 6.9 mmol/l) to a sustainable healthy lifestyle change: ‘*the road map towards diabetes prevention’* (abbreviated: Road Map: RM). This protocol is applied within a primary health care setting by a general practitioner and a practice nurse. The feasibility and (cost-) effectiveness of care provided according to the RM protocol will be evaluated.

**Methods/Design:**

A cluster randomised clinical trial is performed, with randomisation at the level of the general practices. Both opportunistic screening and active case finding took place among clients with high risk factors for diabetes. After IFG is diagnosed, motivated people in the intervention practices receive 3–4 consultations by the practice nurse within one year. During these consultations they are coached to increase the level of physical activity and healthy dietary habits. If necessary, participants are referred to a dietician, physiotherapist, lifestyle programs and/or local sports activities. The control group receives care as usual. The primary outcome measure in this study is change in Body Mass Index (BMI). Secondary outcome measures are waist circumference, physical activity, total and saturated fat intake, systolic blood pressure, blood glucose, total cholesterol, HDL cholesterol, triglycerides and behaviour determinants like risk perception, perceived knowledge and motivation. Based on a sample size calculation 120 people in each group are needed. Measurements are performed at baseline, and after one (post-intervention) and two years follow up. Anthropometrics and biochemical parameters are assessed in the practices and physical activity, food intake and their determinants by a validated questionnaire. The cost-effectiveness is estimated by using the Chronic Disease Model (CDM). Feasibility will be tested by interviews among health care professionals.

**Discussion:**

The results of the study will provide valuable information for both health care professionals and policy makers. If this study shows the RM to be both effective and cost-effective the protocol can be implemented on a large scale.

**Trial registration:**

ISRCTN41209683. Ethical approval number: NL31342.075.10.

## Background

Diabetes is an increasing problem which affects approximately 371 million people worldwide [1]. In 2011 the estimated prevalence in the Netherlands was 834.100 people with diabetes, of which 90% with type 2 diabetes mellitus (T2DM), with an annual estimate of newly discovered cases of 71,000 in that year [2]. This number translates into approximately 5% of the total Dutch population and is expected to rise to 1,300,000 and 1,600,000 in 2025 [3]. Many of these people are not aware of their risk of diabetes, but already have impaired fasting glucose (IFG; according to WHO criteria: 6.1 to 6.9 mmol/l). It is estimated, that some 750.000 people in the Netherlands between 30 and 70 years of age have either IFG or impaired glucose tolerance (IGT) [4]. People with IFG not only have a high risk of developing T2DM on short term, but also have an increased cardiovascular risk [5]. Without changes in lifestyle and proper support, about 9% of people with IFG will develop T2DM within three years [6].

When IFG is diagnosed, follow-up often is not well organized. The Diabetes Mellitus type 2 guideline of the Dutch College of General Practitioners 2006 advices a regular check in people known with IFG for development of T2DM and to treat their cardiovascular risk factors [7]. However, these people are not monitored systematically: most of the times a simple life style advice is given, combined with the advice to have fasting blood glucose measured at regular intervals. Health care providers indicate that there is a need for support in coaching people with IFG in a more systematic and intensive way. They also indicate that they do not know how to handle this group [8].

Lifestyle interventions which focus on both physical activity and diet can be effective for people diagnosed with IFG, with a relative risk reduction of 43% seven years after the start of the intervention [9,10]. Another study shows a similar risk reduction which sustained even 20 years after the start [11,12] Also Dutch projects show that it is possible to stimulate people to change their lifestyle [13-15]. For example, the SLIM study shows an improvement of glucose tolerance, which sustained after three years [16]. While the studies mentioned above show that a health gain can be achieved by people at risk for diabetes, another Dutch study on reducing risk by lifestyle change did not show the expected effects and proved to be not cost-effective [17]. Still it is concluded that combined lifestyle interventions are likely to have great potential as a strategy to prevent diabetes. Actually, these most successful interventions were obtained using many personnel and intensive supervision while the current practice requests less expensive, simple interventions which can be easily carried out in daily practice.

For above mentioned reason and in order to meet the needs of both people at high risk to develop T2DM and health care professionals, the Dutch Diabetes Federation (NDF) developed a protocol for coaching persons with an increased risk of diabetes in general practice called ‘*the road map towards diabetes prevention’* (abbreviated: Road Map: RM). It aims to delay or even prevent the development of T2DM and associated cardiovascular risk factors. The protocol is developed according to the framework of the Diabetes Mellitus type 2 Guideline of the Dutch College of General Practitioners [7]. To include people in the protocol, people at risk for diabetes were stimulated to fill out the Diabetes Risk Test (DRT). The DRT consists of ten questions covering risk factors for diabetes, and provides a score for the probability of developing diabetes within five years [9,18]. This paper describes the design of a RCT into the feasibility and (cost-) effectiveness of care provided according to the RM.

## Methods

### Study design

**Figure 1:**
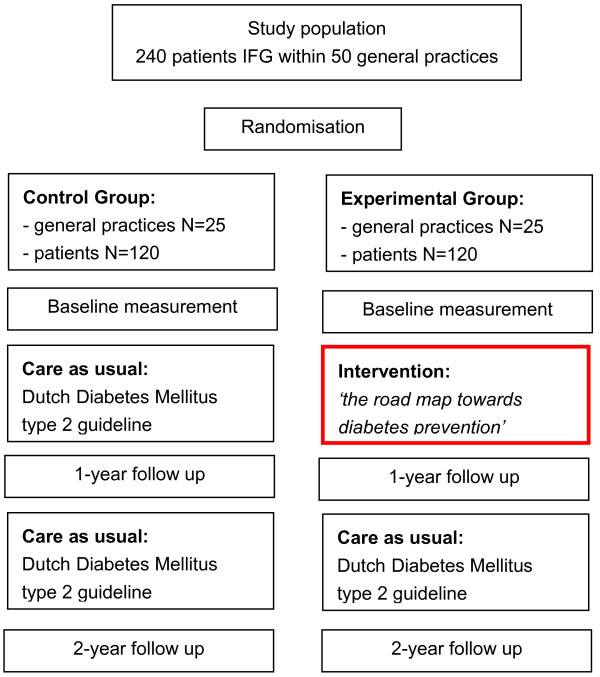
Study design.

A clustered randomized controlled trial is carried out to study the feasibility and (cost-)effectiveness of the RM. Randomization takes place at the level of the general practices. The subjects in the intervention group are treated according to the RM. Persons in the control group receive care as usual, including one consultation each year, according to the T2DM guideline of the Dutch College of General Practitioners [7]. A baseline measurement takes place just after the diagnosis of IFG and just before the start of the intervention. Outcome measurements are performed one year after the diagnosis (t1, 1-year follow-up) and two years after the diagnosis (t2, 2-year follow up) (Figure 1).

### Recruitment of general practices

Recruitment of general practices takes place in the North-eastern region of the Netherlands. Only practices which employ a practice nurse are included. Both, the general practitioner and the practice nurse are responsible for the recruitment and inclusion of participants.

### Recruitment of participants

Potential participants are selected by opportunistic screening and active case finding. Selection criteria for both are the absence of the diagnose diabetes and age (≥45). For opportunistic screening additional selection criteria are weight (BMI ≥ 25) and the presence of cardiovascular risk factors. Opportunistic screening takes place during consultations with the general practitioner and/or practice nurse. Active screening is based on the patient files in the general practices. Selected individuals receive a mailing in which they are asked to do the DRT at home or the DRT is filled out during the consultation. If the DRT shows an increased risk of T2DM, a fasting glucose measure has to be taken.

### Study population

All subjects diagnosed with IFG and motivated to change their lifestyle are included in the study. Exclusion criteria are previous education about IFG or diabetes, emotional, psychological or intellectual problems that are likely to limit the ability of the individual to comply with the protocol, malignant diseases or other diseases or conditions associated with a poor prognosis.

### Training

The practice nurse carries out the intervention with support of the general practitioner, if necessary. Besides carrying out the RM, the practice nurses are responsible for the data collection. Prior to the start of the study, all practice nurses of the intervention group are trained to support them in applying the protocol. Additionally they are trained in techniques of motivational interviewing.

### Randomisation

To avoid contamination of care as usual by the intervention, the randomisation takes place at the level of the general practices. General practices within the same health center are allocated to the same study group. A computerised random number generator is used to allocate the practices to the intervention or the control group.

### Intervention

The RM protocol is based on the ‘stages of change’ model [19]. In this model change is considered as a process directed towards progress through a series of stages (precontemplation, contemplation, preparation, action and maintenance). The education and counselling of an individual persons’ needs depend on the stage he or she is in. People in the precontemplation and contemplation stage require information about the advances of a healthy lifestyle. People in the preparation stage are already aware of the advantages and disadvantages of a healthy lifestyle and require counselling and guidance to improve their ability to change their lifestyle. In the action stage, people do require tips and strategies for making the actual change. In the maintenance stage people may need additional guidance and feedback to stimulate them in maintaining their changed behaviour and to prevent relapse.

**Figure 2:**
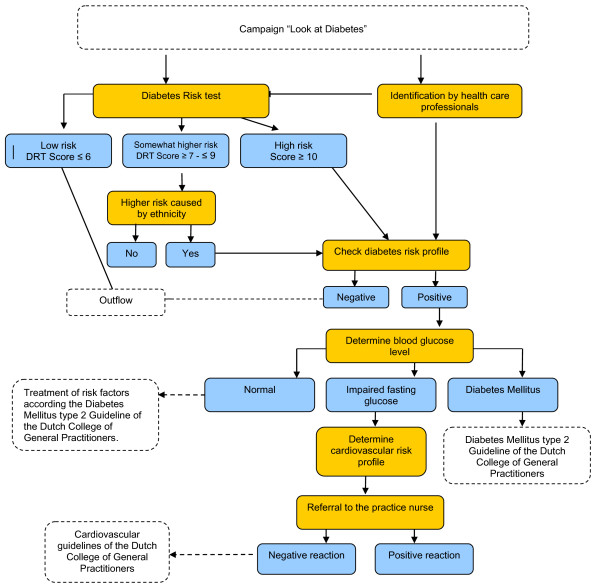
Case finding and testing (first section of the RM protocol).

**Figure 3:**
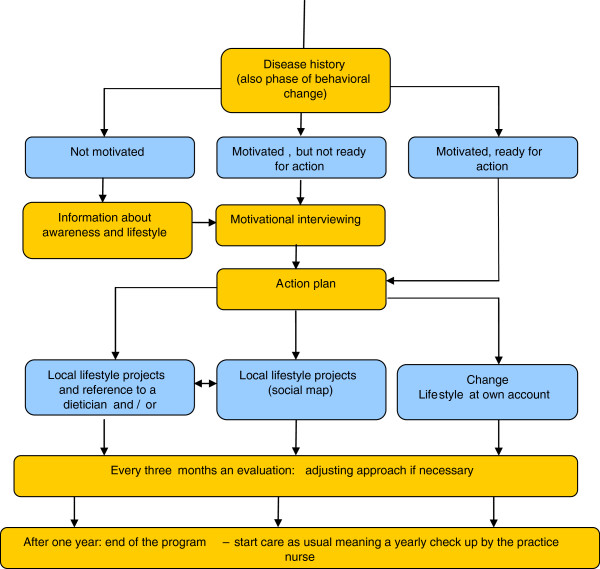
Counselling schedule (second section of the RM protocol).

The first section of the protocol focuses on case finding and testing (Figure 2). The general practitioner is responsible for assessing subjects’ individual risk to develop T2DM. When someone is diagnosed with IFG, the second section of the RM is applied (Figure 3). This section of the protocol focuses on coaching the individual person to a healthy lifestyle. The practice nurse tries to get insight into the motivational situation of the participant. Based on this insight, three to four extra consultations spread over a one year period are carried out. During these consultations the practice nurse gives advice and teaches concrete and applicable skills in order to increase the level of physical activity and healthy dietary habits. If necessary, participants are referred to a dietician, physiotherapist, lifestyle programs and/or local sports activities. The practice nurse applies motivational interviewing techniques to determine someone’s motivational situation and to help a person to change his or her behaviour based on the model of stages of change [19,20]. After one year participants receive care as usual.

After the initial development of the RM protocol it has been piloted on practical feasibility in different small scale pilot studies. The results of these pilots were used to improve the protocol.

### Care as usual

Care as usual for those with IFG means a yearly check on diabetes-related symptoms and blood glucose levels according to the T2DM guideline of the Dutch College of General Practitioners [7].

#### Outcomes

The primary outcome measure in this study is change in Body Mass Index (BMI). Secondary outcome measurements are waist circumference, total and saturated fat intake, physical activity, systolic blood pressure, blood glucose, total cholesterol, triglycerides and the behaviour determinants risk perception, perceived knowledge, motivation, attitude, self efficacy and social norm.

#### Measurements

##### Medical records

The practice nurse measures body weight, blood pressure and waist circumference. Fasting plasma glucose levels, HbA1c, and serum lipids are determined in regional laboratories. Information on family history of diabetes, history of elevated blood glucose, ethnicity, other cardiovascular risk factors and comorbidity are recorded by the practice nurse. Furthermore, the practice nurse is responsible for collecting and reporting this data at three moments in time: just after the diagnosis (t0) and during the yearly follow-up consultations (t1 and t2).

### Questionnaires

All participants are asked to complete a questionnaire at three different time points. The questionnaire is handed over by the practice nurse to the participants right after the diagnosis (t0) and after the yearly follow-up consultations (t1 and t2). Participants send the questionnaire directly to the researchers. The questionnaire at t0 comprises general background information on demographic characteristics (including sex, age, ethnicity, education, household composition and occupational situation). The questionnaires at t0, t1 and t2 comprise general health (including perceived health, smoking behaviour, and alcohol consumption), dietary habits (including total and saturated fat intake) [21] and physical activity (including minutes of light, moderate and intense physical activity per week) [22]. Furthermore, promoting and inhibiting factors for compliance are asked (including appreciation of care regarding to their high risk on diabetes, self-efficacy regarding lifestyle change, attitude towards lifestyle change and social norm on healthy living).

### Process evaluation

A process evaluation is carried out to gather information about the care given in the intervention and control group. This evaluation consists of registration forms, questions in the questionnaire of the intervention group at t1 and t2, and face-to-face interviews with the practice nurses responsible for the study. Time spent on the intervention is recorded on registration forms. In the questionnaires participants in both groups are asked about their experiences with the offered care and referrals. In each participating general practice a face-to-face interview with the practice nurse responsible for the study takes place. During these interviews information is gathered about the inclusion of participants, the implementation of the care, the education and motivation of the practice nurses, the number of referrals, experienced motivation and treatment possibilities of the participants. In the intervention group practice nurse are asked about the perceived effect of the RM and advice for further implementation of the RM.

#### Sample size calculation

The sample size calculation is estimated based on BMI with the objective to detect a decrease in BMI of 0.5 in the intervention group. In the SLIM study, the standard error of the mean in change in BMI was 0.2 kg/m2 and the standard deviation 1.37 [16]. The SLIM study has a comparable population to this study, so it is assumed that the standard error and standard deviations are also comparable. To adjust for clusters, an intra-cluster correlation of 0.011 is used [23]. Furthermore, a power of 80% and a two-sided alpha of 0.05 is used. Based on these assumptions, 120 people in each group are needed.

#### Analysis

##### Effects

Multivariate variance and regression analysis are carried out to determine differences in effect on the primary and secondary outcome measures. All analyses are adjusted for baseline measurements and possible differences between both groups at baseline. To adjust for clustering, multilevel analyses are performed.

### Cost-effectiveness

Costs of the care according to the RM are related to determined effects on BMI and physical activity. The cost-effectiveness is estimated by using the Chronic Disease Model (CDM) developed by the Dutch National Institute for Public Health and the Environment (RIVM) [24-26]. The CDM is a Markov type multi-transition model. The model computes life time health effects, effect on health care costs and costs per quality-adjusted life year (QALY) resulting from the minimum and maximum estimated intervention effect. A cost-effectiveness ratio of less than 20.000 euro per QALY gained is considered cost-effective [27,28].

## Discussion

The aim of the study is to investigate whether the use of the RM adds value (for money) compared to the usual care for people with IFG. The results of the study are used as input in the discussion about integrating lifestyle interventions like the RM in the Dutch health care. The study is carried out in a real life situation: in the daily care by general practices. This means that if effects are found, it is likely these effects of the intervention will sustain when the protocol is implemented on a wider scale.

Thus, the results of the study provide valuable information for both health care professionals and policy makers. When this study proves the RM to be both effective and cost-effective, it will be recommended for all healthcare professionals to integrate protocolled coaching for people with an increased risk of diabetes into daily care in general practice.

## Abbreviations

T2DM: Type 2 diabetes mellitus; IFG: Impaired fasting glucose; NDF: Netherlands Diabetes Federation; RM: Route map diabetes prevention; DRT: Diabetes Risk Test; BMI: Body Mass Index; SQUASH: Short questionnaire to assess health enhancing physical activity; t0: Just after the diagnosis; t1: 1-year follow-up; t2: 2-year follow up; CDM: Chronic Disease Model; RIVM: Dutch National Institute for Public Health and the Environment, QUALY, health care costs and costs per quality-adjusted life year.

## Competing interests

The authors declare that they have no competing interests to disclose.

## Authors’ contributions

RJ, HJGB, IW and GEHMR were responsible for study conceptualization and, design of the study. RJ, HJGB, GEHMR, AH and MM developed the analytic plan. AH drafted the manuscript. MM, HJGB, RJ, GEHMR and IW revised the manuscript. All authors approved the manuscript.

## Pre-publication history

The pre-publication history for this paper can be accessed here:

http://www.biomedcentral.com/1471-2296/14/184/prepub
